# Plasma Aβ42/p-Tau217 ratio and p-Tau217 independently predict CSF-defined Alzheimer’s disease pathology in a Brazilian admixed cohort

**DOI:** 10.3389/fnagi.2026.1773606

**Published:** 2026-04-21

**Authors:** Liara Rizzi, Isadora Cristina Ribeiro, Marjorie Cristina da Rocha Silva, Ítalo Karmann Aventurato, Luis Eduardo Santos, Ananssa Maíra dos Santos Silva, Thaís Lopes Pinheiro, Gustavo Bruniera Peres Fernandes, Fernanda Guarino De Felice, Marcio L. F. Balthazar

**Affiliations:** 1 Laboratory of Imaging and Biomarkers in Cognitive Disorders, School of Medical Sciences, Universidade Estadual de Campinas, Campinas, Brazil; 2 Department of Neurology, School of Medical Sciences, Universidade Estadual de Campinas, Campinas, Brazil; 3 Department of Neuroscience, Mayo Clinic in Florida, Jacksonville, FL, United States; 4 D’Or Institute for Research and Education (IDOR), Rio de Janeiro, Brazil; 5 Clinical Laboratory, Hospital Israelita Albert Einstein, São Paulo, Brazil; 6 Institute of Medical Biochemistry Leopoldo de Meis, Rio de Janeiro, Brazil; 7 Departments of Biomedical and Molecular Sciences, Centre for Neuroscience Studies, Queen’s University, Kingston, ON, Canada; 8 Department of Psychiatry, Queen’s University, Kingston, ON, Canada

**Keywords:** Alzheimer’s disease, β-amyloid, biomarkers, diagnosis, pathology, tau

## Abstract

**Introduction:**

Blood-based biomarkers offer a promising, minimally invasive approach to Alzheimer’s disease (AD) diagnosis, yet validation in admixed populations remains limited. We investigated whether plasma biomarkers predict CSF-defined AD pathology in a Brazilian cohort.

**Methods:**

Seventy-eight older adults [including individuals with mild cognitive impairment (MCI), subjective cognitive decline (SCD), and cognitively unimpaired controls] underwent cognitive testing, neuroimaging, and plasma biomarker assessment. CSF data were available for symptomatic participants (MCI and SCD; *n* = 61), and regression and ROC analyses were performed in the subset with both CSF and APOE genotyping data (*n* = 53). Plasma Aβ42, p-Tau181, p-Tau217, t-Tau, and derived ratios were quantified. Multivariable logistic regression and ROC analyses evaluated prediction of abnormal CSF p-Tau181/Aβ42 and t-Tau/Aβ42, adjusting for age, sex, and APOE ε4 status.

**Results:**

Approximately 25% of individuals with MCI exhibited abnormal CSF p-Tau181/Aβ42 and t-Tau/Aβ42 ratios. Moderate correlations were observed between plasma and CSF biomarkers (r > 0.4), particularly for Aβ42/p-Tau217 and p-Tau217. In adjusted models, plasma p-Tau217 and the Aβ42/p-Tau217 ratio independently predicted abnormal CSF pathology. Each one standard deviation increase in p-Tau217 was associated with 3.53–4.83-fold higher odds of abnormal CSF (*p* ≤ 0.003). In contrast, higher Aβ42/p-Tau217 ratios were associated with substantially lower odds of pathology, with each one standard deviation increase corresponding to a 91%–93% reduction in risk (*p* ≤ 0.002). The ratio showed stronger associations than p-Tau217 alone. ROC analyses demonstrated good discrimination. For CSF p-Tau181/Aβ42, Aβ42/p-Tau217 achieved an AUC of 0.88 (83% sensitivity, 85% specificity), compared with 0.83 for p-Tau217. For CSF t-Tau/Aβ42, both biomarkers yielded AUCs of 0.89.

**Discussion:**

Plasma Aβ42/p-Tau217 and p-Tau217 effectively identify CSF-defined AD pathology in an admixed cohort. While higher p-Tau217 levels were associated with increased odds of pathology, higher Aβ42/p-Tau217 ratios were associated with lower pathological burden and demonstrated stronger effect sizes, supporting the added value of combining amyloid and tau biomarkers. These findings provide initial evidence for local validation of blood-based AD biomarkers in Brazil.

## 1 Introduction

Early identification of Alzheimer’s disease (AD) is important, particularly in pre-dementia stages when emerging therapies may be most effective (Arslan et al., 2024). While cerebrospinal fluid (CSF) analysis and amyloid positron emission tomography (PET) provide key insights into AD pathology, their high cost and invasive nature limit accessibility, especially in low and middle-income countries such as Brazil (Karikari, 2022). Blood-based biomarkers offer a promising, minimally invasive alternative, yet their applicability across admixed populations remains poorly understood (Oliveira et al., 2009).

Alzheimer’s disease frameworks also define the disease as a biological continuum, shifting attention to diagnosis before dementia develops (Jack et al., 2018, 2024). Conditions such as Mild Cognitive Impairment (MCI), characterized by objective cognitive decline with preserved activities of daily living, and Subjective Cognitive Decline (SCD), where individuals report memory concerns despite normal test performance, have gained attention (Albert et al., 2011; Jessen et al., 2020). Reliable biomarkers for these early stages could improve detection and guide intervention.

However, most biomarker studies have been conducted in predominantly Caucasian populations, with limited evaluation in admixed Latin American groups (Gleason et al., 2022; Jack et al., 2018). Brazil’s unique composition - a blend of European, African, Indigenous, and Asian ancestry - underscores the urgent need for inclusive research that reflects global diversity (Moura et al., 2015; Oliveira et al., 2009; Secolin et al., 2019). Accordingly, local validation is essential before plasma biomarkers can be more broadly implemented in clinical practice (Ferreira et al., 2023).

This study evaluates whether plasma biomarkers (Aβ42, p-Tau181, p-Tau217, t-Tau, and their ratios) predict CSF-defined AD pathology in Brazilian individuals with SCD or MCI, when diagnostic uncertainty is greatest. Our findings also provide evidence on minimally invasive diagnostics in an underrepresented population.

## 2 Materials and methods

### 2.1 Participants

The study included 78 individuals aged 55 years or older, comprising 48 with MCI, 13 with SCD, and 17 cognitively unimpaired controls. All participants underwent clinical evaluation, neuropsychological testing, MRI, and plasma biomarker assessment.

Participants were recruited regardless of ethnicity or socioeconomic status and provided written informed consent. Given the geographic location of recruitment, the cohort reflects the admixed Brazilian population, with predominant European ancestry alongside African and Native American contributions, as previously described in regional genetic studies (Secolin et al., 2019). The study was approved by the University of Campinas Research Ethics Committee (CAAE No. 24898619.8.0000.5404) and conducted in accordance with the Declaration of Helsinki.

SCD was defined according to SCD-Initiative (SCD-I) criteria (Jessen et al., 2020), including self-reported cognitive decline lasting at least one year or concerns measured by the Memory Complaint Scale (MCS) (Vale et al., 2012). All SCD participants performed within age- and education-adjusted normative ranges on neuropsychological testing, with no evidence of objective cognitive impairment (i.e., no scores ≤ 1.5 standard deviations below the mean).

Mild cognitive impairment was diagnosed based on NIA-AA criteria (Albert et al., 2011), defined as performance ≥ 1.5 standard deviations below the mean on one cognitive test, or between 1.0 and 1.5 standard deviations below the mean on at least two tests across cognitive domains.

Cognitively unimpaired controls were recruited as community-based volunteers and underwent structured clinical evaluation to confirm the absence of cognitive complaints and neurological or psychiatric conditions. They performed within age- and education-adjusted normative ranges on neuropsychological testing, with no evidence of objective cognitive impairment (i.e., no scores ≤ 1.5 standard deviations below the mean), had preserved functional status (Pfeffer Functional Activities Questionnaire score < 5), and showed no significant abnormalities on neuroimaging.

Exclusion criteria included neurological or psychiatric disorders, uncontrolled systemic diseases (e.g., renal, cardiac, hepatic failure, or uncontrolled diabetes), traumatic brain injury with loss of consciousness, substance abuse, chronic neurotoxin exposure, and severe cerebrovascular disease (Fazekas ≥ 2). Pre-screening laboratory tests included vitamin B12, folate, syphilis, and thyroid function.

Cerebrospinal fluid data were available only for symptomatic participants (MCI and SCD; *n* = 61), as CSF collection in cognitively unimpaired individuals was not approved by the Institutional Ethics Committee due to the absence of clinical indication. Although SCD participants perform within normal ranges on objective testing, they were considered a clinically relevant at-risk group, justifying CSF collection, whereas cognitively unimpaired controls were recruited as community-based volunteers without indication for lumbar puncture. APOE genotyping was available for a subset (*n* = 53) included in regression analyses. Accordingly, all diagnostic accuracy analyses were restricted to participants with available CSF data and were based on CSF biomarker status, independent of clinical diagnosis; cognitively unimpaired controls were included only for descriptive comparisons. Participants were classified as CSF-positive (abnormal CSF biomarker ratios above established cutoffs) or CSF-negative (normal CSF profiles), regardless of clinical grouping (Table 1).

**TABLE 1:** Demographics, clinical assessments, and fluid biomarker levels.

Variable	MCI (*n* = 48)	SCD (*n* = 13)	Controls (*n* = 17)	*P*
Age (years)	66.56 ± 6.69	64.15 ± 6.74	65.11 ± 7.33	NS
Sex (% females)	60.41	76.92^a,c^	58.82	0.024
Education (years)	14.12 ± 4.61	14.23 ± 2.24	15.88 ± 3.35	NS
MoCA	24.27 ± 2.91^a,b^	26.53 ± 1.71	27.12 ± 2.18	<0.001
APOE ε4 (%)	38.46^a,b^	16.66	23.07	0.003
RAVLT A1–A5	−0.51 ± 1.08^a,b^	0.61 ± 0.97	0.70 ± 0.89	<0.001
RAVLT A7	−0.74 ± 1.34^a,b^	0.42 ± 0.82	0.50 ± 1.11	<0.001
RAVLT REC	−0.61 ± 1.44^a,b^	0.60 ± 0.47	0.51 ± 0.75	<0.001
ROCF copy	0.55 ± 0.79 ^a^	1.01 ± 0.32	1.03 ± 0.25	0.009
ROCF immediate	−0.02 ± 1.25^a,b^	0.77 ± 1.08	1.30 ± 1.00	0.002
ROCF delayed	−0.11 ± 1.26^a,b^	0.71 ± 0.92	1.35 ± 1.07	<0.001
TMT-A	−1.39 ± 2.36^a^	0.09 ± 0.74	0.15 ± 1.15	0.007
TMT-B	−0.37 ± 1.37^a,b^	0.93 ± 0.65	0.87 ± 0.79	<0.001
SVF	−0.35 ± 1.11^a^	−0.61 ± 0.78	0.24 ± 0.66	0.030
PVF	−0.32 ± 0.95	−0.29 ± 0.85	0.50 ± 1.41	NS
BNT	−0.92 ± 2.02^a,b^	−0.29 ± 0.85	0.81 ± 1.19	<0.001
CSF Aβ42	948.44 ± 443.50	990.23 ± 340.75	–	NS
CSF p-Tau181	18.11 ± 6.91	15.51 ± 4.76	–	NS
CSF p-Tau231	49.74 ± 48.99	28.48 ± 10.05	–	0.012
CSF t-Tau	208.33 ± 73.84	188.87 ± 55.05	–	NS
Altered CSF p-Tau181/Aβ42 (%)	26.82	8.33	–	<0.001
Altered CSF t-Tau/Aβ42 (%)	24.39	0	–	<0.001
Plasma Aβ42	8.52 ± 1.93	8.99 ± 1.91	9.35 ± 2.43	NS
Plasma t-Tau	2.89 ± 0.90	3.11 ± 1.13	2.94 ± 0.76	NS
Plasma p-Tau181	24.91 ± 15.59	19.70 ± 5.80	22.89 ± 8.27	NS
Plasma p-Tau217	0.165 ± 0.108	0.112 ± 0.04	0.139 ± 0.101	NS
Plasma Aβ42/Aβ40	0.032 ± 0.006	0.037 ± 0.009	0.034 ± 0.003	NS
Plasma Aβ42/t-Tau	0.423 ± 0.186	0.507 ± 0.231	0.438 ± 0.134	NS
Plasma Aβ42/p-Tau181	3.26 ± 1.45	3.16 ± 1.07	3.39 ± 1.28	NS
Plasma Aβ42/p-Tau217	76.54 ± 41.29	92.19 ± 43.02	83.63 ± 32.51	NS

^a^Significantly different from controls.

^b^Significantly different from subjective cognitive decline (SCD).

^c^Significantly different from mild cognitive impairment (MCI). Cerebrospinal fluid (CSF) and plasma biomarker levels in pg/mL. CSF measurements were available only for symptomatic participants (MCI and SCD). Sex, *APOE ε4* status, and altered CSF ratios were analyzed by chi-square test. Continuous variables were assessed using Kruskal-Wallis tests, except age, CSF t-Tau, plasma Aβ42, and p-Tau181, which were analyzed using ANOVA. Values are presented as mean ± SD. Montreal Cognitive Assessment (MoCA) scores are reported as raw values. All other neuropsychological test scores are presented as z-scores, standardized according to normative data, with higher values indicating better performance. For tests in which higher raw scores indicate worse performance [e.g., Trail Making Test (TMT)], values were inverted so that higher z-scores consistently reflect better cognitive performance. NS, not significant.

### 2.2 Neuropsychological assessment

Cognitive function was assessed across multiple domains. Global cognition was evaluated using the Montreal Cognitive Assessment (MoCA) (Apolinario et al., 2018). Episodic memory was assessed using the Rey Auditory Verbal Learning Test (RAVLT) and the delayed recall components of the Rey-Osterrieth Complex Figure Test (ROCF) (Malloy-Diniz et al., 2007). Executive function was examined using the Trail Making Test (TMT-A and TMT-B) (Reitan, 1955). Language abilities were evaluated with the Boston Naming Test (BNT) (Kaplan et al., 1983) and both semantic and phonemic verbal fluency tests (SVF and PVF, respectively) (Epker et al., 1999). Visuospatial skills were assessed using the Rey-Osterrieth Complex Figure Test copy task (ROCF copy) (Osterrieth, 1944).

### 2.3 Magnetic resonance imaging (MRI) evaluation

Magnetic resonance imaging scans were acquired using a Philips 3T Achieva-Intera^®^ scanner. Gray matter analysis used high-resolution 3D T1-weighted images (1 mm isotropic voxels; sagittal plane; matrix: 240 × 240 × 180; TR/TE: 7/3.201 ms; flip angle: 8°). Coronal and axial FLAIR T2-weighted images, aligned to the hippocampus, were used to classify subjects by the Fazekas scale (voxel size: 0.45 × 0.45 × 4.00 mmł; TR/TE/TI: 12,000/140/2,850 ms; FOV: 220 × 206 mm; interslice gap: 1 mm).

### 2.4 Cerebrospinal fluid (CSF) analysis

Cerebrospinal fluid samples from SCD and MCI participants were collected, processed, and stored following standardized protocols (Hansson et al., 2021). Four milliliters of CSF were aliquoted and stored at −80 °C. CSF p-Tau231 was measured using SIMOA HD-X analyzer (Quanterix, Billerica, MA). Aβ42, total tau (t-Tau), and phosphorylated tau at threonine 181 (p-Tau181) were measured using immunoassays on the Roche Cobas^®^ analyzer with Elecsys^®^ kits: Total-Tau CSF, Phospho-Tau (181P) CSF, and β-Amyloid (1-42) CSF II. To assess CSF-defined AD pathology, p-Tau181/Aβ42 and t-Tau/Aβ42 ratios were calculated. Based on Elecsys^®^ guidelines, cut-offs were >0.023 for p-Tau181/Aβ42 and > 0.28 for t-Tau/Aβ42. Values above these thresholds indicate amyloid PET positivity, with >90% concordance (Campbell et al., 2021).

### 2.5 Plasma biomarker analysis

Plasma samples were collected via venipuncture into 4 mL EDTA tubes (BD Vacutainer^®^ K2 or equivalent) and processed within 2 h following standardized protocols (Wu et al., 2025). Aliquots were stored at −80 °C until analysis. Biomarkers were quantified using the SIMOA HD-X analyzer (Quanterix, Billerica, MA), with the Neurology 3-Plex A assay (Aβ1-40, Aβ1-42, t-Tau) and kits for p-Tau181 V2.1, and ALZpath p-Tau217. Calibrators were run in triplicate and controls in duplicate per kit instructions. Prior to analysis, samples were centrifuged at 10,000 *g* for 5 min and analyzed in a single replicate with 4X onboard dilution. Freeze-thaw cycles were limited to two to maintain sample integrity (Verberk et al., 2022).

### 2.6 APOE genotyping

Peripheral blood was collected into 4 mL EDTA tubes (BD Vacutainer^®^ K2 or equivalent), mixed by inversion (8–10 times), and centrifuged at 2,500 rpm for 10 min. Genomic DNA was extracted using the phenol-chloroform-isoamyl alcohol method. Purity and concentration were assessed with a Nanodrop spectrophotometer, ensuring A260/A280 ratios between 1.7–1.9 and A260/A230 > 2.0, indicating minimal contamination. APOE genotyping was performed by real-time PCR using the 7500 System (Thermo Fisher Scientific), targeting SNPs rs429358 and rs7412 to determine APOE alleles (ε2, ε3, ε4).

### 2.7 Statistical analysis

Data normality was assessed with the Shapiro-Wilk test. Group comparisons were conducted for all variables. Neuropsychological test scores (except MoCA) were expressed as z-scores based on age- and education-adjusted normative data and were used as such in group comparisons. Chi-square tests evaluated sex and APOE ε4 status. Normally distributed variables were analyzed using ANOVA or Student’s *t*-test; non-normal variables with Kruskal-Wallis or Mann-Whitney U tests. Spearman’s rank correlation assessed associations between CSF and plasma biomarkers. Logistic regression models were used to evaluate the predictive value of plasma biomarkers for CSF-defined AD pathology (p-Tau181/Aβ42 or t-Tau/Aβ42), adjusting for age, sex, and APOE ε4 status. Analyses were conducted using a complete-case approach, with regression and ROC models restricted to participants with available CSF ratios and APOE genotyping data. p-Tau217 and Aβ42/p-Tau217 were standardized using z-score transformation (subtracting the sample mean and dividing by the sample standard deviation) prior to regression analysis. Accordingly, reported odds ratios reflect the change in odds associated with a one standard deviation increase in the biomarker. ROC curves assessed diagnostic accuracy, and the Youden index identified optimal cut-offs. *p* < 0.05 was considered significant. Analyses were performed in SPSS version 22 (SPSS Inc., Chicago, IL, United States).

## 3 Results

### 3.1 Participant characteristics

Baseline demographics, neuropsychological tests, and fluid biomarker levels are summarized in Table 1. No significant differences were observed between the groups in terms of age or schooling. However, the proportion of females was higher in the SCD group. As expected, global cognition (MoCA) was significantly higher in controls compared to individuals with SCD and MCI. Across neuropsychological domains, individuals with MCI showed significantly worse performance than both SCD and controls, particularly in episodic memory (RAVLT and ROCF delayed recall), executive function (TMT), and language (BNT). The prevalence of the APOE *ε4* allele was higher in the MCI group. Absolute CSF biomarker levels were similar across groups, except for p-Tau231. However, the proportion of individuals with abnormal CSF ratios was significantly higher in MCI. When applying Elecsys^®^ cut-offs for altered CSF (p-Tau181/Aβ42 > 0.023 and t-Tau/Aβ42 > 0.28), the MCI group showed about 25% of altered CSF p-Tau181/Aβ42 and t-Tau/Aβ42 ratios.

Magnetic resonance imaging was performed in all participants to support clinical characterization and to exclude alternative causes of cognitive impairment, such as significant cerebrovascular disease, tumors, or other structural abnormalities. Neuroimaging findings were not used as inclusion criteria or analytical variables, but rather as part of the clinical assessment to ensure appropriate diagnostic classification and exclusion of confounding conditions.

### 3.2 Correlations between plasma and CSF biomarkers

**FIGURE 1:**
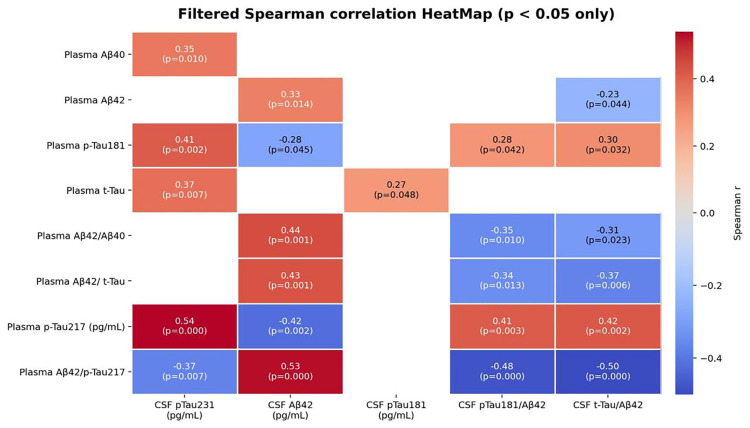
Spearman correlation heatmap between cerebrospinal fluid (CSF) and plasma biomarkers. Heatmap illustrating significant Spearman correlations (*p* < 0.05) between plasma biomarkers (Aβ40, Aβ42, p-Tau181, t-Tau, p-Tau217, and derived ratios) and CSF biomarkers (Aβ42, p-Tau181, p-Tau231, p-Tau181/Aβ42, and t-Tau/Aβ42). Correlation coefficients (r) and corresponding *p*-values are displayed within each cell. Red indicates positive correlations and blue indicates negative correlations, with color intensity proportional to the strength of association. Only statistically significant correlations are shown.

**FIGURE 2:**
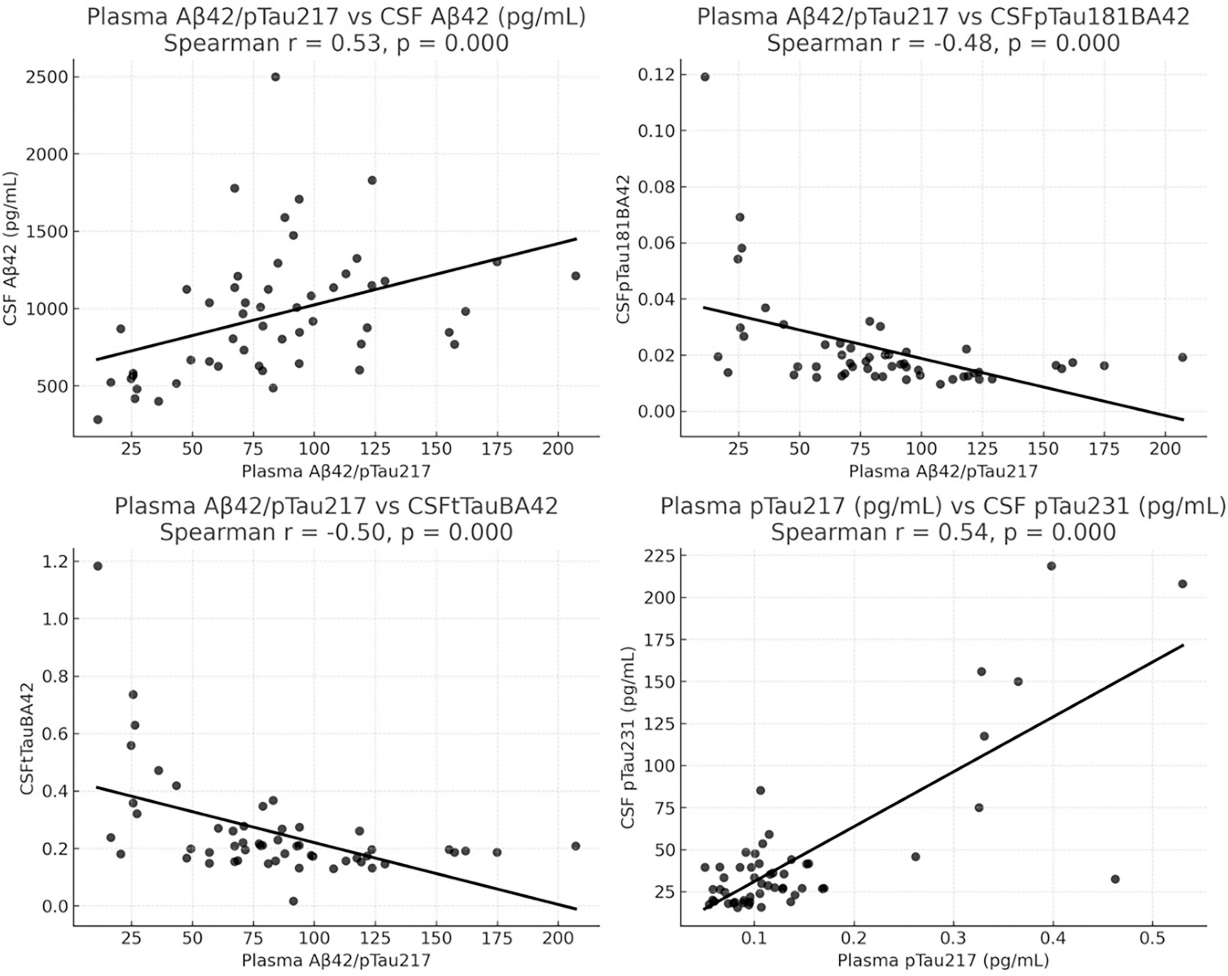
Top four significant Spearman correlations between plasma and CSF biomarkers (*p* < 0.05). Scatter plots illustrating the four strongest correlations between plasma and CSF biomarkers. Top left shows plasma Aβ42/p-Tau217 ratio vs. CSF Aβ42 (*r* = 0.53, *p* < 0.001). Top right shows plasma Aβ42/p-Tau217 ratio vs. CSF p-Tau181/Aβ42 (*r* = –0.48, *p* < 0.001). Bottom left shows plasma Aβ42/p-Tau217 ratio vs. CSF t-Tau/Aβ42 (*r* = –0.50, *p* < 0.001). Bottom right shows plasma p-Tau217 vs. CSF p-Tau231 (*r* = 0.54, *p* < 0.001). Lines represent fitted linear regression trends for visualization purposes.

Spearman’s rank correlation analysis revealed moderate correlations (r > 0.4) between CSF and plasma biomarkers. All CSF and plasma biomarkers, including their respective ratios, were assessed. Significant correlations are illustrated in Figure 1. Notably, plasma Aβ42/p-Tau217 showed moderate correlations with CSF Aβ42 (*r* = 0.53), CSF p-Tau181/Aβ42 (*r* = −0.48), and CSF t-Tau/Aβ42 (*r* = −0.50). Plasma p-Tau217 also correlated with CSF p-Tau231 (*r* = 0.54) (Figure 2). Plasma Aβ42/Aβ40 and plasma Aβ42/t-Tau were moderately correlated with CSF Aβ42 (*r* = 0.44 and *r* = 0.43, respectively). Additionally, plasma p-Tau181 showed moderate correlations with CSF p-Tau231/Aβ42 (*r* = 0.43) and CSF p-Tau231 (*r* = 0.41). These findings suggest that plasma biomarkers reflect underlying CSF pathology, supporting their potential utility as accessible indicators of CSF alterations within the central nervous system.

### 3.3 Plasma biomarkers predicting CSF-defined AD pathology

We performed four logistic regression analyses to evaluate the predictive value of plasma biomarkers for CSF-defined AD pathology, defined by CSF biomarker status (abnormal p-Tau181/Aβ42 or t-Tau/Aβ42 ratios). Analyses were restricted to participants with available CSF and APOE genotyping data, and plasma p-Tau217 and the Aβ42/p-Tau217 ratio were standardized prior to modeling.

For abnormal CSF p-Tau181/Aβ42, plasma p-Tau217 remained independently associated with CSF pathology after adjustment for age, sex, and APOE ε4 status. Each one standard deviation increase in plasma p-Tau217 was associated with a 3.53-fold higher odds of abnormal CSF (95% CI 1.55–8.00, *p* = 0.003). In the model including plasma-derived ratios, only the Aβ42/p-Tau217 ratio remained significant. Higher values of this ratio were associated with lower odds of abnormal CSF pathology. Specifically, each one standard deviation increase in the ratio was associated with an approximately 91% reduction in the odds of abnormal CSF (OR = 0.09, 95% CI 0.02–0.39, *p* = 0.001).

For abnormal CSF t-Tau/Aβ42, plasma p-Tau217 was independently associated with CSF pathology, with each one standard deviation increase corresponding to a 4.83-fold higher odds of abnormal CSF (95% CI 1.87–12.50, *p* = 0.001). In the model including plasma-derived ratios, the Aβ42/p-Tau217 ratio remained the only significant predictor, with each one standard deviation increase associated with a 93% reduction in the odds of abnormal CSF (OR = 0.07, 95% CI 0.01–0.36, *p* = 0.002).

### 3.4 Plasma Aβ42/p-Tau217 ratio and p-Tau217 reflect CSF-defined AD pathology

**FIGURE 3:**
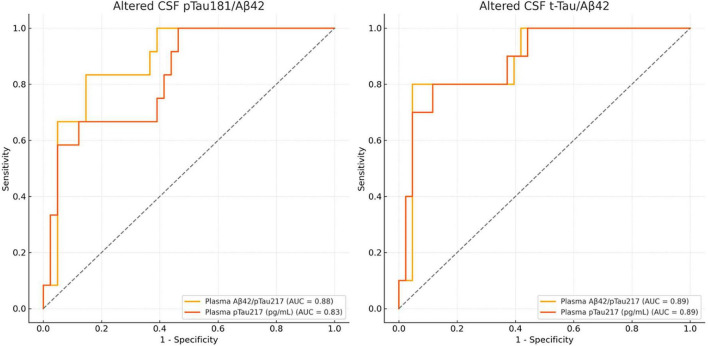
Receiver operating characteristic (ROC) curves for plasma Aβ42/p-Tau217 and p-Tau217 in detecting cerebrospinal fluid (CSF)-defined Alzheimer’s disease (AD) pathology. **(Left)** For abnormal CSF p-Tau181/Aβ42, the Aβ42/p-Tau217 ratio demonstrated superior discrimination (AUC = 0.88; sensitivity = 83.3%; specificity = 85.4%; cutoff = 66.6) compared with p-Tau217 alone (AUC = 0.83; sensitivity = 66.7%; specificity = 87.8%; cutoff = 0.152). **(Right)** For abnormal CSF t-Tau/Aβ42, both biomarkers achieved good performance (AUC = 0.89). The Aβ42/p-Tau217 ratio (cutoff = 43.4) yielded 70% sensitivity and 95.3% specificity, whereas p-Tau217 (cutoff = 0.152) showed 80% sensitivity and 88.4% specificity.

Receiver operating characteristic (ROC) curve analyses were conducted to evaluate the performance of plasma Aβ42/p-Tau217 and plasma p-Tau217 in detecting CSF-defined AD pathology (Figure 3).

For abnormal CSF p-Tau181/Aβ42, the Aβ42/p-Tau217 ratio demonstrated good discrimination, with an area under the curve (AUC) of 0.88 (95% CI 0.75–0.98). Using a cutoff of 66.6, lower values of the ratio were associated with abnormal CSF, yielding 83.3% sensitivity and 85.4% specificity (PPV = 62.5%, NPV = 94.6%). Plasma p-Tau217 showed slightly lower performance (AUC = 0.83, 95% CI 0.69–0.96); using a cutoff of 0.152, higher values were associated with abnormal CSF pathology, yielding 66.7% sensitivity and 87.8% specificity (PPV = 61.5%, NPV = 90%).

For abnormal CSF t-Tau/Aβ42, both biomarkers demonstrated similar discrimination (AUC = 0.89). The Aβ42/p-Tau217 ratio, using a cutoff of 43.4, showed 70% sensitivity and 95.3% specificity (PPV = 77.8%, NPV = 93.2%), with lower ratio values indicating abnormal CSF. Plasma p-Tau217 (cutoff = 0.152), where higher values indicate abnormal CSF pathology, achieved 80% sensitivity and 88.4% specificity (PPV = 61.5%, NPV = 95%).

## 4 Discussion

This cross-sectional study shows that plasma Aβ42/p-Tau217 and p-Tau217 reliably identify CSF-defined Alzheimer’s disease (AD) pathology in Brazilian individuals with SCD and MCI, independent of clinical diagnosis. These findings support their utility as accessible, minimally invasive screening biomarkers in pre-dementia stages and provide novel evidence in an admixed population. This is, to our knowledge, the first study evaluating plasma-based predictors of CSF-defined AD pathology in a Brazilian cohort.

Among the biomarkers evaluated, plasma p-Tau217 and particularly the Aβ42/p-Tau217 ratio showed the most consistent associations with CSF-defined pathology. While higher p-Tau217 levels were associated with increased odds of AD pathology, higher Aβ42/p-Tau217 ratios were strongly associated with lower pathological burden. Notably, the ratio demonstrated larger effect sizes than p-Tau217 alone, supporting the added value of integrating amyloid and tau information. Prior studies show that p-Tau217 alone demonstrates high diagnostic accuracy for amyloid and tau pathology (Ashton et al., 2024; Barthélemy et al., 2024). However, our results further support evidence that combining amyloid and tau measures improves performance (Lehmann et al., 2025b; Piura et al., 2025). Niimi et al. (2024) reported improved prediction of Aβ-PET positivity when combining plasma Aβ and p-Tau217. Wang et al. (2025) found that the inverse ratio p-Tau217/Aβ42 outperformed p-Tau217 alone, especially in community-based cohorts. Similarly, Lehmann et al. (2025a) observed better diagnostic performance for the p-Tau217/Aβ42 ratio over individual markers. While other combinations, such as Aβ42/Aβ40, have also been proposed (Palmqvist et al., 2024), our data suggest that the Aβ42/p-Tau217 ratio alone provides robust discrimination, extending these findings to a Brazilian cohort.

In line with the regression findings, ROC analyses showed that both biomarkers achieved good diagnostic performance, with the Aβ42/p-Tau217 ratio showing higher specificity and consistently high negative predictive values. Importantly, lower ratio values and higher p-Tau217 levels were associated with abnormal CSF pathology, consistent with their expected biological directionality. The high negative predictive values observed in this cohort further support the potential utility of these biomarkers, particularly the ratio, as minimally invasive rule-out tools in clinical settings. The diagnostic performance observed in our study (AUC 0.88–0.89) aligns with earlier studies (Barthélemy et al., 2024; Brickman et al., 2021; Olvera-Rojas et al., 2025). However, cutoffs should be interpreted strictly as derivation-cohort estimates and not as clinically applicable thresholds, as their performance is likely to be attenuated in independent external populations (Kang et al., 2023). Emerging approaches suggest that flexible or multi-threshold frameworks may be more appropriate than fixed cutoffs, depending on clinical context (Giacomucci et al., 2025; Schindler et al., 2024; Verberk et al., 2024).

Beyond its clinical relevance, this study provides data from a Brazilian admixed cohort, a population that has been largely overlooked in AD biomarker research. Most prior work has been conducted in predominantly Caucasian cohorts from Europe, North America, and Australia (Karikari, 2022), despite known differences in AD risk, biomarker profiles, and disease expression across populations. Brazil’s highly admixed population highlights the need for local validation, as biomarker performance may vary with genetic ancestry and environmental factors. Expanding research in such populations is critical for ensuring equitable clinical implementation (Moura et al., 2015; Oliveira et al., 2009; Santos et al., 2025; Secolin et al., 2019). Diversity in research populations is essential for a comprehensive understanding of AD, as disease presentation and biomarker profiles vary across genetic, environmental, and sociocultural contexts (Babulal et al., 2019; Knell et al., 2025). Such differences have been observed across neuroimaging, neuropsychological measures, CSF, and blood-based biomarkers (Lennon et al., 2022; Weiner et al., 2023). Notably, Black/African American and Hispanic/Latino individuals have a disproportionately higher risk of AD, approximately 2- and 1.5-fold higher, respectively, yet remain underrepresented in research (Lim et al., 2023). Existing evidence highlights variability in amyloid burden, CSF thresholds, and plasma biomarker performance across racial and ethnic groups, with most reference standards derived from predominantly non-Hispanic White cohorts (Arslan et al., 2024; Karikari, 2022; Xiong et al., 2024). Despite this, minority participation in clinical trials remains limited, as illustrated by Lecanemab studies, where these groups comprised only about 20% of participants (Pittock et al., 2023). Together, these disparities underscore the need for validation of AD biomarkers in diverse and admixed populations.

From a clinical perspective, plasma biomarkers could reduce reliance on costly and invasive methods such as PET and CSF analysis, improving access to early diagnosis and facilitating patient selection for disease-modifying therapies (Hansson et al., 2023; Mattsson-Carlgren and Palmqvist, 2023). Our focus on SCD and MCI is particularly relevant, as these stages represent a window where diagnostic uncertainty is highest and intervention may be most effective.

This study has several strengths, including standardized protocols, state-of-the-art platforms (SIMOA and Elecsys^®^), and comprehensive clinical and biomarker characterization. However, limitations should be considered. The relatively small sample size may limit generalizability and increase the risk of overfitting. The absence of CSF or PET data in cognitively normal controls limits comparisons across the full clinical spectrum and prevents exclusion of preclinical AD pathology in this group. Importantly, our study was designed to evaluate plasma biomarkers as predictors of CSF-defined AD pathology within a biological framework, rather than to discriminate between clinically defined groups. Strict inclusion and exclusion criteria improved internal validity but may reduce applicability to more medically complex, real-world populations. Plasma cutoffs were derived and tested within the same cohort and therefore require external validation. Additionally, the CSF p-Tau181/Aβ42 and t-Tau/Aβ42 ratios used as reference standards are imperfect and may involve about 10% misclassification, potentially affecting performance estimates. Although adjusted for age, sex, and APOE ε4 status, other genetic and environmental factors were not assessed. The cross-sectional design also precludes evaluation of longitudinal trajectories and disease progression. Larger, independent, and longitudinal studies in more diverse cohorts are needed to validate these findings and refine clinical thresholds.

In summary, plasma Aβ42/p-Tau217 and p-Tau217 are promising minimally invasive biomarkers for detecting underlying AD pathology in pre-dementia stages within an admixed population. These findings support their potential as scalable screening tools in clinical practice and research, expanding equitable access to AD care and clinical trials, particularly in regions where access to established diagnostic methods is limited.

## Data Availability

The raw data supporting the conclusions of this article will be made available by the authors, without undue reservation.
