# Correction: Plasma Aβ42/p-Tau217 ratio and p-Tau217 independently predict CSF-defined Alzheimer's disease pathology in a Brazilian admixed cohort

**DOI:** 10.3389/fnagi.2026.1875333

**Published:** 2026-06-09

**Authors:** Liara Rizzi, Isadora Cristina Ribeiro, Marjorie Cristina da Rocha Silva, Ítalo Karmann Aventurato, Luis Eduardo Santos, Ananssa Maíra dos Santos Silva, Thaís Lopes Pinheiro, Gustavo Bruniera Peres Fernandes, Fernanda Guarino De Felice, Marcio L. F. Balthazar

**Affiliations:** 1 Laboratory of Imaging and Biomarkers in Cognitive Disorders, School of Medical Sciences, Universidade Estadual de Campinas, Campinas, Brazil; 2 Department of Neurology, School of Medical Sciences, Universidade Estadual de Campinas, Campinas, Brazil; 3 Department of Neuroscience, Mayo Clinic in Florida, Jacksonville, FL, United States; 4 D'Or Institute for Research and Education (IDOR), Rio de Janeiro, Brazil; 5 Clinical Laboratory, Hospital Israelita Albert Einstein, São Paulo, Brazil; 6 Institute of Medical Biochemistry Leopoldo de Meis, Rio de Janeiro, Brazil; 7 Departments of Biomedical and Molecular Sciences, Centre for Neuroscience Studies, Queen's University, Kingston, ON, Canada; 8 Department of Psychiatry, Queen's University, Kingston, ON, Canada

**Keywords:** Alzheimer's disease, β-amyloid, biomarkers, diagnosis, pathology, tau

In the published article, in Table 1, the labels referring to plasma p-Tau181 and plasma t-Tau were inadvertently interchanged. Similarly, the labels for plasma Aβ42/p-Tau181 and plasma Aβ42/t-Tau were also inadvertently interchanged. The corrected Table 1 appears below.

**Table 1:** Demographics, clinical assessments, and fluid biomarker levels.

Variable	MCI (*n* = 48)	SCD (*n* = 13)	Controls(*n* = 17)	*p*
Age (years)	66.56 ± 6.69	64.15 ± 6.74	65.11 ± 7.33	NS
Sex (% females)	60.41	76.92^a^^,^^c^	58.82	0.024
Education (years)	14.12 ± 4.61	14.23 ± 2.24	15.88 ± 3.35	NS
MoCA	24.27 ± 2.91^a^^,^^b^	26.53 ± 1.71	27.12 ± 2.18	<0.001
APOE ε4 (%)	38.46^a^^,^^b^	16.66	23.07	0.003
RAVLT A1–A5	−0.51 ± 1.08^a^^,^^b^	0.61 ± 0.97	0.70 ± 0.89	<0.001
RAVLT A7	−0.74 ± 1.34^a^^,^^b^	0.42 ± 0.82	0.50 ± 1.11	<0.001
RAVLT REC	−0.61 ± 1.44^a^^,^^b^	0.60 ± 0.47	0.51 ± 0.75	<0.001
ROCF Copy	0.55 ± 0.79^a^	1.01 ± 0.32	1.03 ± 0.25	0.009
ROCF Immediate	−0.02 ± 1.25^a^^,^^b^	0.77 ± 1.08	1.30 ± 1.00	0.002
ROCF Delayed	−0.11 ± 1.26^a^^,^^b^	0.71 ± 0.92	1.35 ± 1.07	<0.001
TMT-A	−1.39 ± 2.36^a^	0.09 ± 0.74	0.15 ± 1.15	0.007
TMT-B	−0.37 ± 1.37^a^^,^^b^	0.93 ± 0.65	0.87 ± 0.79	<0.001
SVF	−0.35 ± 1.11^a^	−0.61 ± 0.78	0.24 ± 0.66	0.030
PVF	−0.32 ± 0.95	−0.29 ± 0.85	0.50 ± 1.41	NS
BNT	−0.92 ± 2.02^a^^,^^b^	−0.29 ± 0.85	0.81 ± 1.19	<0.001
CSF Aβ42	948.44 ± 443.50	990.23 ± 340.75	–	NS
CSF p-Tau181	18.11 ± 6.91	15.51 ± 4.76	–	NS
CSF p-Tau231	49.74 ± 48.99	28.48 ± 10.05	–	0.012
CSF t-Tau	208.33 ± 73.84	188.87 ± 55.05	–	NS
Altered CSF p-Tau181/Aβ42 (%)	26.82	8.33	–	<0.001
Altered CSF t-Tau/Aβ42 (%)	24.39	0	–	<0.001
Plasma Aβ42	8.52 ± 1.93	8.99 ± 1.91	9.35 ± 2.43	NS
Plasma t-Tau	2.89 ± 0.90	3.11 ± 1.13	2.94 ± 0.76	NS
Plasma p-Tau181	24.91 ± 15.59	19.70 ± 5.80	22.89 ± 8.27	NS
Plasma p-Tau217	0.165 ± 0.108	0.112 ± 0.04	0.139 ± 0.101	NS
Plasma Aβ42/Aβ40	0.032 ± 0.006	0.037 ± 0.009	0.034 ± 0.003	NS
Plasma Aβ42/t-Tau	0.423 ± 0.186	0.507 ± 0.231	0.438 ± 0.134	NS
Plasma Aβ42/p-Tau181	3.26 ± 1.45	3.16 ± 1.07	3.39 ± 1.28	NS
Plasma Aβ42/p-Tau217	76.54 ± 41.29	92.19 ± 43.02	83.63 ± 32.51	NS

^a^Significantly different from controls.

^b^Significantly different from subjective cognitive decline (SCD).

^c^Significantly different from mild cognitive impairment (MCI).

Cerebrospinal fluid (CSF) and plasma biomarker levels in pg/mL. CSF measurements were available only for symptomatic participants (MCI and SCD). Sex, *APOE* ε*4* status, and altered CSF ratios were analyzed by chi-square test. Continuous variables were assessed using Kruskal-Wallis tests, except age, CSF t-Tau, plasma Aβ42, and p-Tau181, which were analyzed using ANOVA. Values are presented as mean ± SD. Montreal Cognitive Assessment (MoCA) scores are reported as raw values. All other neuropsychological test scores are presented as z-scores, standardized according to normative data, with higher values indicating better performance. For tests in which higher raw scores indicate worse performance [e.g., Trail Making Test (TMT)], values were inverted so that higher z-scores consistently reflect better cognitive performance. NS, not significant.

In the published article, in Figure 1, the labels referring to plasma p-Tau181 and plasma t-Tau were inadvertently interchanged.

The corrected Figure 1 appears below.

**Figure 1:**
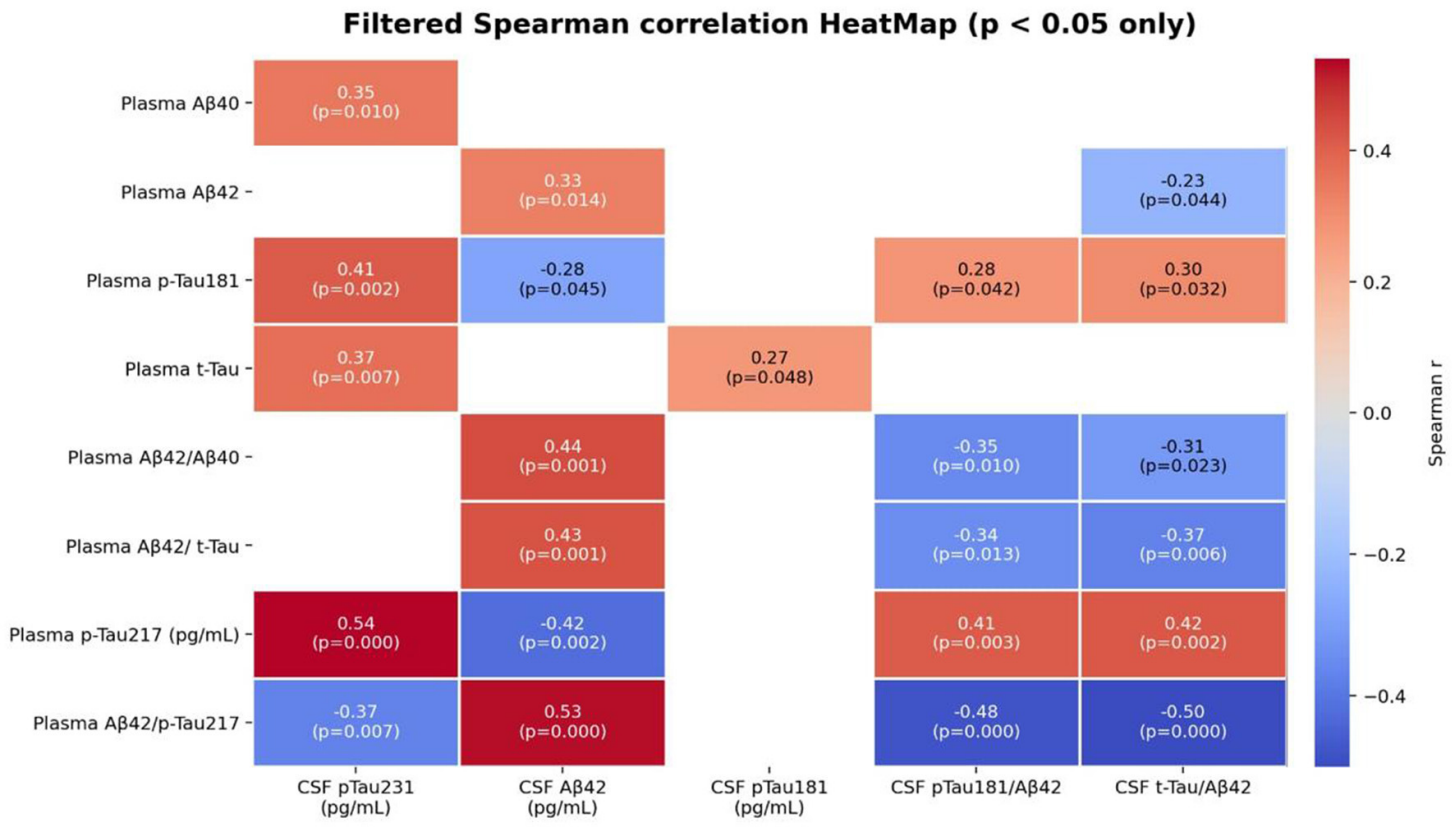
Spearman correlation heatmap between cerebrospinal fluid (CSF) and plasma biomarkers. Heatmap illustrating significant Spearman correlations (*p* < 0.05) between plasma biomarkers (Aβ40, Aβ42, p-Tau181, t-Tau, p-Tau217, and derived ratios) and CSF biomarkers (Aβ42, p-Tau181, p-Tau231, p-Tau181/Aβ42, and t-Tau/Aβ42). Correlation coefficients (*r*) and corresponding *p*-values are displayed within each cell. Red indicates positive correlations and blue indicates negative correlations, with color intensity proportional to the strength of association. Only statistically significant correlations are shown.

In the published article, in the section **3.2 Correlations between plasma and CSF biomarkers**, the labels for plasma p-Tau181 and plasma t-Tau were inadvertently interchanged. The corrected sentence appears below.

“Plasma Aβ42/Aβ40 and plasma Aβ42/t-Tau were moderately correlated with CSF Aβ42 (*r* = 0.44 and *r* = 0.43, respectively). Additionally, plasma p-Tau181 showed moderate correlations with CSF p-Tau231/Aβ42 (*r* = 0.43) and CSF p-Tau231 (*r* = 0.41).”

The original version of this article has been updated.

